# Observational study of the outcomes and costs of initiating maintenance therapies in patients with moderate exacerbations of COPD

**DOI:** 10.1186/1465-9921-13-41

**Published:** 2012-05-31

**Authors:** Anand A Dalal, Manan B Shah, Anna O D’Souza, Orsolya E Lunacsek, Saurabh P Nagar, Glenn D Crater

**Affiliations:** 1 GlaxoSmithKline, Research Triangle Park, 5 Moore Dr, Bide West, Mail Stop B.3153, Durham, NC 27709, USA; 2 Xcenda, 4114 Woodlands Parkway, Suite 500, Palm Harbor, FL 34685, USA

**Keywords:** Chronic obstructive pulmonary disease, Moderate COPD exacerbations, Costs, Anticholinergics, Fluticasone propionate-salmeterol combination

## Abstract

**Background:**

There are limited data describing patients with moderate COPD exacerbations and evaluating comparative effectiveness of maintenance treatments in this patient population. The study examined COPD patients with moderate COPD exacerbations. COPD-related outcomes were compared between patients initiating fluticasone propionate-salmeterol 250/50 mcg (FSC) vs anticholinergics (ACs) following a moderate COPD exacerbation.

**Methods:**

This retrospective observational study used a large administrative claims database (study period: 2003–2009) to identify and describe patients with an initial, moderate COPD exacerbation. A descriptive analysis of patients with moderate COPD exacerbations was done evaluating maintenance treatment rates, subsequent COPD exacerbation rates, and COPD-related costs during a 1-year period. A cohort analysis compared COPD exacerbation rates and associated costs during a variable-length follow-up period between patients initiating maintenance therapy with FSC or ACs. COPD exacerbations were reported as rate per 100 patient-years, and monthly costs were reported (standardized to USD 2009). COPD exacerbation rates between cohorts were evaluated using Cox proportional hazards models, and costs were analyzed using generalized linear models with log-link and gamma distribution.

**Results:**

21,524 patients with a moderate COPD exacerbation were identified. Only 25% initiated maintenance therapy, and 13% had a subsequent exacerbation. Annual costs averaged $594 per patient. A total of 2,849 treated patients (FSC = 925; AC = 1,924) were eligible for the cohort analysis. The FSC cohort had a significantly lower rate of COPD exacerbations compared to the AC cohort (20.8 vs 32.8; *P* = 0.04). After adjusting for differences in baseline covariates, the FSC cohort had a 42% significantly lower risk of a COPD exacerbation (HR = 0.58; 95% CI: 0.38, 0.91). The FSC cohort incurred significantly higher adjusted pharmacy costs per patient per month by $37 (95% CI: $19, $72) for COPD-related medications vs the AC cohort. However, this increase was offset by a significant reduction in adjusted monthly medical costs per patient for the FSC vs the AC cohort ($82 vs $112; *P* < 0.05). Total monthly COPD-related costs, as a result, did not differ between cohorts.

**Conclusions:**

Only a quarter of patients with a moderate COPD exacerbation were subsequently treated with maintenance therapy. Initiation of FSC among those treated was associated with better clinical and economic outcomes compared to AC.

## Background

Chronic obstructive pulmonary disease (COPD) is cha-racterized by limited and irreversible airflow and accompanied by a range of pathological changes in the lung [1]. COPD is associated with significant mortality, as the Centers for Disease Control and Prevention reported chronic lower respiratory diseases to be the third-leading cause of death in the United States (US) in 2009, accounting for 44.7 deaths per 100,000 persons [2].

In addition to its significant mortality, COPD carries a substantial economic burden. In the US in 2010, the total costs of COPD were estimated at $49.9 billion, with $29.5 billion attributed to direct healthcare expenditures. Hospitalizations accounted for the largest portion of direct costs at $13.2 billion, followed by $5.8 billion in prescription drug costs, and $5.5 billion in physician services [3]. Not surprisingly, COPD costs are directly related to the severity of disease [4,5]. A study of commercial insurance claims data from the US showed the cost of a severe exacerbation to be 2.6 times greater than the cost of a non-severe exacerbation [5].

The Global Initiative for Chronic Obstructive Lung Disease (GOLD) provides a staging system for catego-rizing severity of stable COPD, and defines stages for mild, moderate, severe, and very severe disease [1]. Moderate COPD is the most commonly diagnosed stage, comprising 46% to 54% of all patients with COPD [6-8], and is defined using spirometry as forced expiratory volume in one second (FEV_1_)/forced vital capacity (FVC) <0.70 and 50% ≤ FEV_1_ <80% predicted. It is at the moderate stage that patients typically present for me-dical care, due to persisting respiratory symptoms or an acute exacerbation [1]. For patients with moderate COPD, maintenance therapy with bronchodilators (inhaled long-acting beta-agonists [LABAs] and anticholinergics [ACs]) is recommended [1]. The addition of inhaled corticosteroid (ICS)-containing drugs is recommended for patients with repeated COPD exacerbations or those with severe COPD [1]. A post-hoc analysis of a large, randomized controlled trial (TORCH) has shown that initiation of maintenance pharmacotherapy with an ICS-containing drug at earlier disease stages (moderate vs severe) can potentially modify disease progression in COPD, and reduce exacerbation rates compared to placebo [9]. Post-hoc analysis of the TORCH study in a subset of patients with moderate COPD as defined by the GOLD stage II (≥50% FEV_1_) showed that treatment with ICS-containing drugs (combination of fluticasone propionate-salmeterol, or fluticasone alone) significantly reduced the rate of COPD exacerbations by 31% (95% confidence interval [CI]: 19, 40) compared to placebo.

Preliminary evidence from the TORCH study suggests a benefit of using ICS-containing drugs in patients with moderate COPD. However, large randomized trials powered to analyze the moderate COPD population are required to provide definitive answers. In the absence of clinical trial data, we designed a study to evaluate the impact of specific maintenance therapies for patients with a moderate COPD exacerbation using administrative claims data. Absence of lung function parameters (FEV_1_ and FVC) precluded assessment of COPD severity, and accordingly, patients with moderate COPD could not be evaluated, and instead patients with a moderate COPD exacerbation were evaluated. The current study had a 2-fold purpose: 1) to characterize patients with moderate COPD exacerbations, and 2) to evaluate differential COPD exacerbation rates and related costs between patients initiating maintenance therapy with fluticasone propionate-salmeterol combination 250/50 mcg (FSC) or ACs after an initial moderate COPD exacerbation.

## Methods

### Data source

Data from January 1, 2003 to March 31, 2009 were extracted from the IMS LifeLink Health Plan Claims Database for this study. The Database contains data from over 90 different managed healthcare plans encompassing over 60 million lives. The database includes inpatient and outpatient diagnoses using International Classification of Diseases, 9^th^ Revision, Clinical Modification (ICD-9-CM) codes, procedures using Current Procedural Terminology (CPT) codes and Healthcare Common Procedure Coding System (HCPCS) codes, and both standard and mail-order prescription records. The payer type distribution for this data source is 80% commercial, 3% Medicaid, and less than 2% Medicare Risk, with the remainder categorized as “other.” The dataset is de-identified, and hence research conducted with such data is exempt from ethical approval and is only required to be compliant with the Health Insurance Portability and Accountability Act.

### Sample selection and study design

The study population was composed of patients with a moderate COPD exacerbation who had not pre-viously received maintenance therapy. Additionally, patients were screened to ensure they did not have a COPD-related (ICD-9-CM codes of chronic bronchitis [491.xx], emphysema [492.xx], and chronic airway obstruction [496.xx]) hospitalization or emergency department (ED) visit (medical claim with a primary discharge diagnosis for a hospitalization or primary diagnosis for ED visit) in the year before their index moderate COPD exacerbation. Using a previously defined algorithm [10,11], a moderate COPD exacerbation was characterized as a physician visit with a primary diagnosis of COPD (ICD-9-CM codes 491.xx, 492.xx, or 496.xx) with a prescription for an oral corticosteroid (OCS) or antibiotic within 5 days of the visit. This definition may have captured exacerbations that would *clinically* be defined as mild; however, the events were classified as moderate since the patient experienced symptoms requiring them to seek medical attention. The date of the first moderate COPD exacerbation was defined as the index date for each patient. Maintenance therapies included ICS mono-therapy, LABA monotherapy, ICS+LABA combination therapy, and ACs (including tiotropium and ipratropium alone or in fixed combination with albuterol).

**Figure 1:**
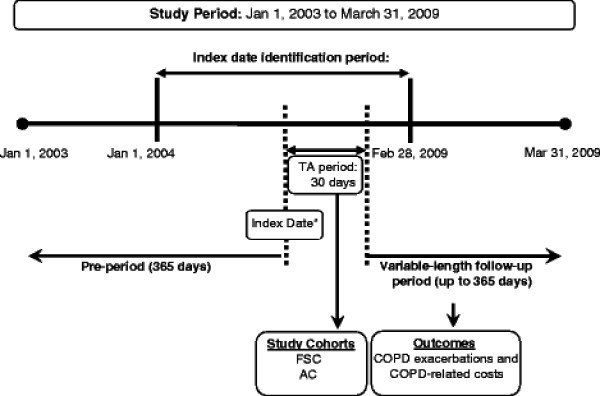
**Study design - cohort analysis. *KEY: ****AC* – anticholinergic; *COPD* – chronic obstructive pulmonary disease; *FSC* – fluticasone propionate-salmeterol 250/50 mcg; *TA* – treatment assessment. ^a^ Index date was the date of the first moderate exacerbation, which was defined as a physician outpatient visit with a primary diagnosis of COPD and included an oral corticosteroid or antibiotic prescription within 5 days of the physician outpatient visit.

The study was done in 2 phases to evaluate the objectives. Phase 1 comprised a descriptive analysis to characterize patients with moderate COPD exacerbations and evaluated treatment patterns, exacerbation rates, and COPD-related healthcare utilization and costs during a 1-year period after the index date. A retrospec-tive cohort design was employed in Phase 2 to evaluate the impact of initiation of select COPD maintenance therapies on outcomes. The cohort study design ensured temporality of receipt of maintenance therapy to the outcomes studied. Patients were eligible for the cohort analysis if they received a study maintenance therapy within 30 days of their index date (treatment assessment period) (Figure 1). The 2 cohorts were named FSC and AC, (which included tiotropium and ipratropium alone or in fixed combination with albuterol) depen-ding on the first drug received during the treatment assessment period (index drug). A variable-length follow-up period was adopted for the cohort analysis, where patients were followed until:

• They switched (defined as a switch to any COPD-related maintenance medication different from the index drug); or

• They discontinued their study medication (defined as having more than a 60-day gap between the end of the days’ supply of the preceding prescription and the fill date of the next consecutive prescription); or

• The end of continuous eligibility; or

• The end of the study period; or

• A maximum follow-up of 1 year was reached

The length of the variable follow-up period could therefore range from 1 day to 1 year after the 30-day treatment assessment period.

Patients with a moderate COPD exacerbation who were aged 40 years or older on their index date and had continuous health plan eligibility 1 year prior to the index date (pre-period) were eligible for inclusion for both the descriptive and cohort analyses. For the descriptive analysis, all patients were required to have continuous eligibility for the 1-year period after the index date as well. Patients were excluded from both the descriptive and cohort analyses if they had received maintenance therapy (as defined earlier) in the pre-period, had a diagnosis of COPD during the pre-period (ie, a diagnosis code in the primary field of 491.xx, 492.xx, or 496.xx), or if they had an exclusionary comorbid medical condition (cystic fibrosis [277.0x], bronchiectasis [494.xx], respiratory cancer [160–163.xx, 231.xx], pulmonary fibrosis [515.xx], pneumoconiosis [500–505.xx], sarcoidosis [135.xx], or pulmonary tuberculosis [011.xx]) anytime during the pre- and follow-up periods.

### Outcome measures and statistical analyses

A descriptive analysis was done to profile patients with a moderate exacerbation in terms of their rate and frequency of COPD exacerbations, COPD-related costs, and maintenance treatment rates during the 1-year period after the index date. COPD exacerbations were defined as a COPD-related hospitalization or ED visit or a physician visit with an OCS or antibiotic prescription within 5 days of the visit. COPD-related costs were valued in 2009 US dollars (USD) and were estimated using the allowed payment for all COPD-related medical and pharmacy services. The allowed payment was chosen because it best represented the actual amount that the provider was eligible to receive from all parties, including third-party payers and patients. COPD-related medical services included medical claims with a primary diagnosis of COPD and hospitalization claims with a primary discharge diagnosis of COPD, while COPD-related prescriptions included ICS, OCS, LABAs, short-acting beta-agonists (SABAs), ACs, xanthines, fixed-dose combinations, and antibiotics.

In the cohort analysis, outcomes and costs were compared between the FSC and AC cohorts. COPD exacerbations, as previously defined, were reported as a rate per 100 person-years of follow-up due to the variability in follow-up time of patients. Differences between cohorts in time to COPD exacerbations were evaluated using Cox proportional hazards models controlling for potential confounders identified in the pre-period, including: age, region, number of SABA canisters, number of OCS prescriptions, home oxygen therapy, physician specialty on the index date, comorbid asthma or upper respiratory tract infection or lower respiratory tract infection, and Charlson comorbidity index (CCI) score. COPD-related costs, as previously defined, were also compared between the FSC and AC cohorts. Adjusted costs and differences in costs were assessed using gene-ralized linear models (GLMs) with log-link and gamma distribution controlling for the aforementioned variables, as well as pre-period COPD-related pharmacy costs (log-transformed).

All statistical tests performed tested a 2-sided hypothesis of no difference between groups at a significance level of 0.05. Analyses were conducted with SAS software (version 9.1.3 for Windows; SAS Institute, Cary, NC).

## Results

There were 82,749 patients identified with a moderate COPD exacerbation, and 21,524 patients met the study selection criteria. Lack of continuous eligibility for medical and pharmacy services in the pre-period was the primary reason for exclusion from the study (35% of the initial sample identified). Demographic characteristics for the total study population for the descriptive analysis and the FSC and AC cohorts for the cohort analysis are shown in Table 1.

**Table 1:** Baseline description of study sample

	**Descriptive analysis**	**Cohort analysis**	
**Characteristic**	**TOTAL N = 21,524**	**FSC n = 925**	**AC n = 1,924**
**Age, mean (SD)**	57.4 (11.2)	57.0 (10.2)	59.2^a^ (10.6)
**Female, n (%)**	12,220 (56.8%)	557 (60.2%)	1,066^a^ (55.4%)
**US geographic region,**^**a**^**n (%)**			^a^
**East**	7,213 (33.5%)	367 (39.7%)	498 (25.9%)
**Midwest**	7,603 (35.3%)	320 (34.6%)	772 (40.1%)
**South**	3,250 (15.1%)	138 (14.9%)	324 (16.8%)
**West**	3,458 (16.1%)	100 (10.8%)	330 (17.2%)
**CCI, mean (SD)**	0.81 (1.3)	0.87 (1.4)	0.84 (1.3)
**Asthma, n (%)**	2,054 (9.5%)	156 (16.9%)	209^a^ (10.9%)
**URTI, n (%)**	6,607 (30.7%)	298 (32.2%)	520^a^ (27.0%)
**LRTI, n (%)**	4,937 (22.9%)	287 (31.0%)	545 (28.3%)
**Number of SABA canisters, mean (SD)**	0.39 (1.8)	0.86 (2.8)	0.52^a^ (1.9)
**Number of OCS prescriptions, mean (SD)**	0.35 (1.2)	0.42 (1.2)	0.41 (1.2)
**Use of home oxygen therapy, n (%)**	167 (0.8%)	6 (0.6%)	35^a^ (1.8%)
**COPD-related pharmacy costs,**^**b**^**mean (SD)**	$85 (165)	$102 (160)	$83^a^ (171)
**Pulmonologist/allergist care on index date, n (%)**	1,072 (5.0%)	86 (9.3%)	114^a^ (5.9%)

***KEY*: ***AC* – anticholinergic; *CCI* – Charlson comorbidity index; *COPD* – chronic obstructive pulmonary disease; *FSC* – fluticasone propionate-salmeterol 250/50 mcg; *OCS* – oral corticosteroid; *LRTI* – lower respiratory tract infection; *SABA* – short-acting beta agonist; *SD* – standard deviation; *URTI* – upper respiratory tract infection.

^a^Significant difference between FSC and AC cohorts at level 0.05; all other comparisons are non-significant.

^b^Pharmacy costs only include costs of non-maintenance medications, as no maintenance medications are allowed in the pre-period.

### Descriptive analysis

Patients with an index moderate COPD exacerbation had an average age of 57 years, with a slightly higher proportion of females (Table 1). The chronic comorbid burden at baseline was minimal, as noted by a CCI of less than 1, and only ~10% of the cohort had concomitant asthma. Almost a quarter of patients, however, had previously experienced a respiratory tract infection. During the pre-period, use of rescue medications was less than one canister per month, as was reflected in the low annual pharmacy costs ($85).

During the 1-year follow-up period, 25.3% (n = 5,445) of patients received treatment with a maintenance therapy following their index moderate COPD exacerbation (Table 2). A total of 12.8% (n = 2,751) of the study population had a subsequent exacerbation of any severity level, and the mean number of COPD exacerbations per patient was 0.18. Patients admitted to the hospital or ED for their exacerbation comprised 1.9% of the total population, or 15.1% of the 2,751 with a subsequent exacerbation. Total average annual COPD-related costs for the study population were $594 per patient, with costs being composed of slightly greater medical costs than pharmacy costs.

**Table 2:** Descriptive analysis: outcomes in 1-year follow-up period

**Outcome**	**Total N = 21,524**
**Maintenance treatment rate, n (%)**	5,445 (25.3%)
**COPD exacerbation rate (overall), n (%)**	2,751 (12.8%)
**Requiring hospitalization/ED visit**	417 (1.9%)
**Number of COPD exacerbations (overall), mean (SD)**	0.18 (0.57)
**Requiring hospitalization/ED visit**	0.02 (0.15)
**Total COPD-related cost per patient, mean (SD) (pharmacy + medical)**	$594 (1,626)
**COPD-related pharmacy costs**	$293 (542)
**COPD-related medical costs**	$301 (1,431)

***KEY*: ***COPD* – chronic obstructive pulmonary disease; *ED* – emergency department; *SD* – standard deviation.

### Cohort analysis

A total of 2,849 patients met the selection criteria for the cohort analysis; 925 in the FSC cohort and 1,924 in the AC cohort (73.6% ipratropium and 26.4% tiotropium). The 2 cohorts varied in several demographic characteristics (Table 1) and trended toward more advanced COPD in the FSC cohort, including significantly higher prevalence of comorbid asthma, rescue medication use, and use of pulmonologist or allergist care. The mean duration of follow-up was significantly longer in the FSC compared to the AC cohort (88 days vs 75 days; *P* < 0.01).

**Figure 2:**
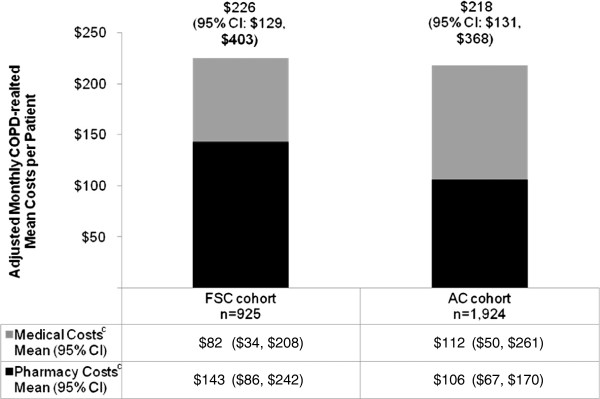
**Adjusted monthly COPD-related mean costs per patient by drug cohort**^**a,b**^**. ***KEY: AC* – anticholinergic; *CI* – confidence interval; *COPD* – chronic obstructive pulmonary disease; *FSC* – fluticasone propionate-salmeterol 250/50 mcg. ^a^Each cost component was estimated from separate regression models and represents a predicted, rather than an observed value. Pharmacy and medical costs may not sum to total. Costs are in 2009 USD. ^b^Costs obtained from generalized linear models with log-link and gamma distribution controlling for age, region, Charlson comorbidity index score, number of short-acting beta-agonist canisters, number of oral corticosteroid prescriptions, comorbid asthma or upper respiratory tract infection or lower respiratory tract infection, home oxygen therapy, physician specialty on the index date, and pre-period COPD-related pharmacy costs (logged). ^c^Significant difference between FSC and AC (referent group) cohorts at alpha level 0.05.

During the follow-up period, the FSC cohort had a 42% significantly lower risk of any COPD exacerbation (adjusted hazard ratio [HR] = 0.58; 95% CI: 0.38, 0.91) compared to the AC cohort (Table 3). The unadjusted rate of having any COPD exacerbation per 100 person-years was 20.8 for the FSC cohort vs 32.8 for the AC cohort. Only 2.2 and 7.2 COPD exacerbations per 100 person-years required hospitalization or an ED visit in the FSC and AC cohorts, respectively; the risk of hospitalization or ED visit was 77% lower in the FSC cohort (adjusted HR = 0.23; 95% CI: 0.07, 0.84). The total monthly adjusted COPD-related costs per patient did not differ significantly between the 2 cohorts (mean difference = $8; 95% CI: -$2, $35) (Figure 2). Pharmacy costs per patient per month were significantly higher in the FSC cohort (mean difference = $37; 95% CI: $19, $72; *P* < 0.05), whereas medical costs per patient per month were significantly lower in the FSC cohort (mean difference = −$30; 95% CI: -$16, -$53; *P* < 0.05).

**Table 3:** Exacerbations by drug cohort

**Outcome**	**FSC cohort N = 925**	**AC cohort N = 1,924 (Reference group)**	**Unadjusted *P*-value**	**Adjusted HR and *P*-value**^**a **^**(95% CI)**
**COPD exacerbation rate per 100 person-years, n**	20.8	32.8	*P* = 0.04	0.58 *P* = 0.02 (0.38, 0.91)
** Requiring hospitalization /ED visit**	2.2	7.2	*P* = 0.06	0.23 *P* = 0.03 (0.07, 0.84)

***KEY*: ***AC* – anticholinergic; *CI* – confidence interval; *COPD* – chronic obstructive pulmonary disease; *FSC* – fluticasone propionate-salmeterol 250/50; *HR* – hazard ratio.

^a^Adjusted HRs obtained from Cox proportional hazards model controlling for age, region, Charlson comorbidity index score, number of short-acting beta-agonist cans, number of oral corticosteroid prescriptions, comorbid asthma or upper respiratory tract infection or lower respiratory tract infection, home oxygen therapy, and physician specialty on the index date.

## Discussion

In this study, 1 in 4 patients initiated maintenance therapy with AC, ICS+LABA, ICS monotherapy, or LABA monotherapy following a moderate COPD exacerbation. Most studies evaluating drug therapy rates in the COPD population have included use of SABAs across all severity levels, precluding a comparison with our study [12,13]. However, a recent study by Fitch et al. evaluated drug therapy patterns by severity levels using a claims-based algorithm, and found that 26% of patients with moderate COPD in a commercially insured population were prescribed a long-acting bronchodilator [8]. Unlike Fitch et al., our study analyzed patients with a moderate COPD exacerbation, and not necessarily those with moderate COPD disease, because spirometry information is not captured in administrative data. Another caveat is that Fitch’s estimate excludes ICS-containing products and ipratropium, the short-acting AC. In contrast, the current study includes ipratropium, suggesting that the use of only long-acting bronchodilators may be even lower than what was observed in this study.

The above data indicate possible under-treatment in a COPD population experiencing a moderate COPD exacerbation. However, it is also plausible that, despite experiencing a moderate COPD exacerbation, these patients were sufficiently ‘mild’ in severity not to have required maintenance treatment, and maintenance treatment was then reserved for those with subsequent COPD exacerbations. However, of the 13% of patients who had a subsequent exacerbation in this study following their index moderate COPD exacerbation, only 48% received maintenance therapy, suggesting sub-optimal treatment of the COPD population.

Because one of the objectives of maintenance pharmacotherapy is preventing COPD exacerbations, therapies that most effectively prevent COPD exacerbations have the potential to improve outcomes and decrease costs. Although there are studies that compare changes in lung function between maintenance therapy classes for patients with moderate COPD [14-16], there are limited data evaluating exacerbation outcomes outside of a clinical trial setting for this COPD subset. There is preliminary information, albeit in post-hoc analyses, from 2 large trials comparing maintenance therapies with placebo that have shown a significant reduction in the COPD exacerbation rate for patients with moderate COPD [9,17]. However, evidence around head-to-head comparisons is lacking.

In the absence of comparative clinical trial data, observational studies using retrospectively collected data permit evaluation of comparative treatments and provide for robust sample sizes. This observational study provides a valuable evidence base to differentiate among available maintenance therapies—specifically, the combination of FSC and the AC agents that include tiotropium. This study demonstrated that patients receiving maintenance therapy with FSC had a 42% significantly lower risk of exacerbation than patients taking ACs. Despite the differences in COPD exacerbations between cohorts, the total monthly cost did not differ between cohorts. Further examination of the component medical and pharmacy costs revealed that the FSC cohort had significantly higher pharmacy costs, whereas the AC cohort had significantly higher medical costs. Therefore, the lower COPD exacerbation rate in the FSC cohort did result in lower medical costs; however, any cost-savings were offset by higher drug-acquisition costs. This population-based study using actuarial claims data provides much-needed data isolating the costs and impact of 2 maintenance therapy options.

The study design warrants specific discussion of both strengths and limitations. Because the study used claims data, lung function data were not available for use in classifying the severity of the underlying pre-exacerbation COPD of the patients enrolled in this study. Therefore, it is possible that patients with moderate exacerbation have varying COPD severity levels, and do not necessarily include patients with moderate COPD. Although this is a notable limitation, baseline demographic characteristics of the study sample are consistent with that of patients with moderate COPD exacerbations, providing validation for this classification method. The inclusion criteria strengthened the study design by excluding patients who had previously received maintenance therapy and those with a previous COPD-related hospitalization or ED visit. Patients with asthma were not excluded from the study sample because of the high overlap of comorbid asthma and COPD and the challenges associated with discerning between the two conditions without clinical data. The adjustment of baseline differences to obtain valid drug cohort comparisons can be considered to have been done correctly to the extent that appropriate variables were used to capture the characteristics. Adjustment in this study is only limited by the ability to capture these characteristics accurately, and residual confounding may exist if the underlying severity is not adequately captured by these proxy measures, especially unobservable confounders. However, the direction of the results shows a low likelihood of any selection bias, as the FSC cohort was more severe than the AC cohort at baseline.

Misclassification bias may exist in any claims-based study due to miscoding or under-coding of claims. However, the likelihood of this bias in the present study is low, as all outcomes required a primary diagnosis of COPD, where the probability of miscoding is low due to reimbursement requirements. The outcome of a moderate COPD exacerbation was defined as a physician visit with a diagnosis of COPD plus an OCS or antibiotic within 5 days. The requirement of drug therapy within 5 days has been previously used [10,11], is conservative, and increases the likelihood that the medication was associated directly with the COPD-related physician visit. The proportion of patients with an exacerbation who also had a hospitalization or ED visit was also reported, but could not be stratified by the proportion of patients in the intensive care unit (which would indicate a more severe exacerbation), due to the small sample size of hospitalized patients. The study design applied variable lengths of follow-up to preserve sample sizes, and the time-dependent model allowed for a completer rather than an intent-to-treat analysis. This type of an analysis also allows for censoring at the time of treatment switch or discontinuation of the index medication, thus ensuring that the outcome is related to the exposure. Finally, results of this study are generalizable only to a specific subset of COPD patients enrolled in a commercial managed care population, and the inclusion criteria applied to increase the internal validity of this study may reduce the generalizability of its findings.

## Conclusions

Use of maintenance therapy in patients with moderate COPD exacerbations is less than optimal. This study provides data to differentiate costs and outcomes between 2 maintenance therapy options, FSC and ACs. Initiation of FSC is associated with a significantly lower risk of subsequent COPD exacerbations, without an increase in total costs, compared to ACs in moderate COPD exacerbations. These data provide useful information for prescribers and health policy decision makers in the selection of maintenance therapies that reduce COPD exacerbations, without increasing COPD-related medical costs. Further research should seek to validate these findings using lung function data, in order to determine the clinical severity level of the patient population.

## Abbreviations

AC: Anticholinergic; COPD: Chronic obstructive pulmonary disease; CCI: Charlson comorbidity index; ED: Emergency department; FEV_1_: Forced expiratory volume in one second; FSC: Fluticasone propionate-salmeterol 250/50 mcg; FVC: Forced vital capacity; GOLD: Global Initiative for Chronic Obstructive Lung Disease; HR: Hazard ratio; LABA: Long-acting beta-agonist; LRTI: Lower respiratory tract infection; SABA: Short-acting beta-agonist; SD: Standard deviation; URTI: Upper respiratory tract infection; US: United States; USD: United States dollars; CI: Confidence interval; CPT: Current Procedural Terminology; GLM: Generalized linear model; HCPCS: Healthcare Common Procedure Coding System; ICD-9-CM: International Classification of Diseases, 9th Revision, Clinical Modification; ICS_1_: Inhaled corticosteroid; OCS: Oral corticosteroid.

## Competing interests

This study was sponsored by GSK (study protocol number ADC113901). AD Dalal and GD Crater are employees of GlaxoSmithKline (GSK) and own company stock. MB Shah, AO D’Souza and OE Lunacsek are employees of Xcenda, LLC, a company that received funding from GSK to conduct this research. SP Nagar works in a contractor role within GSK and obtains compensation from GSK.

## Authors’ contributions

AAD, MBS, SPN, GDC, and AOD have made substantial contributions to conception and design, acquisition of data, drafting the article or revising it critically for important intellectual content, and final approval of the version to be published; AAD, MBS, SPN, AOD, and OEL have made substantial contributions to analysis and interpretation of data. SPN had a key role in providing the initial datacut for analytic file creation, obtaining the data, and sending it to Xcenda. All authors have read and approved the final manuscript.
